# DNA supercoiling-induced shapes alter minicircle hydrodynamic properties

**DOI:** 10.1093/nar/gkad183

**Published:** 2023-03-27

**Authors:** Radost Waszkiewicz, Maduni Ranasinghe, Jonathan M Fogg, Daniel J Catanese, Maria L Ekiel-Jeżewska, Maciej Lisicki, Borries Demeler, Lynn Zechiedrich, Piotr Szymczak

**Affiliations:** Institute of Theoretical Physics, Faculty of Physics, University of Warsaw, Pasteura 5, 02-093 Warsaw, Poland; University of Lethbridge, Dept. of Chemistry and Biochemistry, Alberta, T1K3M4, Canada; Department of Molecular Virology and Microbiology, Verna and Marrs McLean Department of Biochemistry and Molecular Biology, Department of Pharmacology and Chemical Biology, Baylor College of Medicine, One Baylor Plaza, Houston, TX 77030, USA; Department of Biosciences, Rice University, 6100 Main St., Houston, TX 77005-1827, USA; Institute of Fundamental Technological Research, Polish Academy of Sciences, A. Pawińskiego 5B, 02-106 Warsaw, Poland; Institute of Theoretical Physics, Faculty of Physics, University of Warsaw, Pasteura 5, 02-093 Warsaw, Poland; University of Lethbridge, Dept. of Chemistry and Biochemistry, Alberta, T1K3M4, Canada; University of Montana, Department of Chemistry and Biochemistry, Missoula, MT 59812, USA; Department of Molecular Virology and Microbiology, Verna and Marrs McLean Department of Biochemistry and Molecular Biology, Department of Pharmacology and Chemical Biology, Baylor College of Medicine, One Baylor Plaza, Houston, TX 77030, USA; Institute of Theoretical Physics, Faculty of Physics, University of Warsaw, Pasteura 5, 02-093 Warsaw, Poland

## Abstract

DNA in cells is organized in negatively supercoiled loops. The resulting torsional and bending strain allows DNA to adopt a surprisingly wide variety of 3-D shapes. This interplay between negative supercoiling, looping, and shape influences how DNA is stored, replicated, transcribed, repaired, and likely every other aspect of DNA activity. To understand the consequences of negative supercoiling and curvature on the hydrodynamic properties of DNA, we submitted 336 bp and 672 bp DNA minicircles to analytical ultracentrifugation (AUC). We found that the diffusion coefficient, sedimentation coefficient, and the DNA hydrodynamic radius strongly depended on circularity, loop length, and degree of negative supercoiling. Because AUC cannot ascertain shape beyond degree of non-globularity, we applied linear elasticity theory to predict DNA shapes, and combined these with hydrodynamic calculations to interpret the AUC data, with reasonable agreement between theory and experiment. These complementary approaches, together with earlier electron cryotomography data, provide a framework for understanding and predicting the effects of supercoiling on the shape and hydrodynamic properties of DNA.

## INTRODUCTION

Nearly seventy years after Rosalind Franklin’s meticulous work that led to the first description of the structure of DNA (1), we are still working to understand how this remarkable molecule is organized, stored, activated, and segregated into daughter cells (2–9). It is becoming increasingly apparent that negative supercoiling (the underwinding of the DNA double helix) provides a secondary or ‘hidden code’ that contributes to the three-dimensional (3-D) organization of the genome (10) that can be used by cells as a ‘molecular servomechanism’ to detect and regulate gene expression (11).

We recently discovered that the degree of curvature, dictated by DNA loop length, additionally tunes supercoiling-mediated effects and promotes mechanical crosstalk to expose DNA bases at specific distant sites (12). Exposed DNA bases drastically increase DNA flexibility to change the 3-D structure of DNA, which, conversely, influences the location and frequency of the disruptions to base pairing (12). Therefore, 3-D shape and base exposure are manifestations of supercoiling and looping (13). These findings underscore how supercoiling-dependent conformational changes may allow DNA to be an active participant in its transactions (13,14).

We previously used minicircles of a few hundred base pairs and defined supercoiling to determine how supercoiling and looping modulates the 3-D structure of DNA (13). DNA loops in this length range are found in nature, e.g., wrapped around the nucleosome, and are involved in gene regulation (15–18). Human topoisomerase IIα relaxes supercoiled DNA minicircles (13), demonstrating that they are biologically active. Thus, minicircles are biologically relevant and mimic DNA loops naturally occurring in cells.

We previously determined how supercoiling modulates the shapes of minicircles using electron cryotomography (cryoET) (13). Although informative, these assays are laborious and only limited DNA sequences and buffer conditions have been explored (12,13,19). CryoET provides 3-D information on individual DNA minicircles of defined supercoiling (DNA topoisomers) (13), but the approach requires skill in the art, is time-consuming and the resulting structures are of low resolution. Increasingly powerful, atomic force microscopy (AFM) can show the helical repeat of DNA as well as areas of base pair disruption (19), but the sequence is unidentifiable and the technique requires that DNA is adsorbed onto a flat surface. Not only might this adsorption distort DNA conformations but it means that they are visualized as 2-D projections with limited 3-D information.

Computational modeling would be of great value in helping predict DNA negative supercoiling and looping behavior, but thus far fails to account for supercoiling- and looping-mediated site-specific base exposure or resulting conformational changes. For example, most efforts at understanding looping (20) or cyclizing of DNA (21) ignore supercoiling. Therefore, new modeling efforts are needed, including the parameters of supercoiling and degree of curvature (dictated by DNA loop length). Before this modeling can be improved, however, detailed parameters of supercoiled loops of DNA must be determined.

Whereas cryoET is impractical to use for multiple conditions, AUC and electrophoresis rapidly assess properties in solution, and can be used to test multiple conditions simultaneously. Toward the goal of understanding how DNA sequence and 3-D shape are affected by negative supercoiling and looping, here we combined state-of-the-art AUC (22) with mathematical modeling to determine hydrodynamic parameters of supercoiled DNA minicircles. We derived partial specific volume (PSV) and anisotropy, and measured the sedimentation and diffusion coefficient for minicircle DNA of different degrees of negative supercoiling and lengths. With these values, we determined the density of DNA. We discovered that DNA length and supercoiling strongly affect the sedimentation properties of minicircle DNA but have either no or only a minimal effect on the PSV.

We generalized the continuum elastic framework to accurately predict the previously observed DNA minicircle 3-D shapes (13). This generalization provides additional and complimentary information that will allow us to interpret supercoiling- and curvature-dependent DNA structural alterations. Emboldened by this accomplishment, we then combined the measured elastic and hydrodynamic properties of DNA minicircles using bead models and considering force and hydrodynamic effects to compute the hydrodynamic sedimentation and diffusion coefficients. These modeling results compared favorably to AUC measurements.

## MATERIALS AND METHODS

### Chemicals and reagents

MseI, Nb.BbvCI, Proteinase K, T4 DNA Ligase, low molecular weight DNA ladder, and 100 bp DNA ladder were purchased from New England Biolabs (Ipswich, MA, USA). Adenosine triphosphate (ATP), antifoam 204, dithiothreitol (DTT), ethidium bromide, and RNase A were purchased from Sigma-Aldrich (St. Louis, MO, USA). Acrylamide, ampicillin, chloroform, and sodium chloride were purchased from Fisher Scientific (Pittsburgh, PA, USA). All other chemicals were purchased from VWR International (West Chester, PA, USA).

### Generation and purification of minicircle DNA

Plasmid pMC336 (13) was used to generate both the 336 bp and 672 bp minicircles via λ-integrase-mediated site-specific recombination as described (23). Double-length 672 bp minicircles contain two copies of the 336 bp minicircle sequence in tandem orientation and are generated by the recombination used to generate 336 bp minicircle DNA.

### Generation of different DNA topologies

The ‘supercoiled’ samples are the 336 bp or 672 bp minicircle products of the purification process. These were analyzed without further manipulation. To make nicked DNA, the minicircles were nicked at a single site using the nicking endonuclease Nb.BbvCI according to the manufacturer’s protocol. The 672 bp minicircle contains two copies of the BbvCI site and was thus nicked at both locations. Following nicking, the DNA was subsequently incubated at 80 ℃ for 20 minutes to inactivate Nb.BbvCI. Linear 336 bp was generated by incubating supercoiled 336 bp minicircle with MseI according to the manufacturer’s protocol. The linearized DNA was subsequently incubated at 65 ℃ for 20 minutes to inactivate the enzyme. ‘Relaxed’ 336 bp minicircle DNA was generated by incubating the nicked minicircles with T4 DNA ligase in 50 mM Tris-Cl pH 7.5, 10 mM MgCl_2_, 1 mM ATP, and 10 mM DTT overnight at room temperature. ‘Hypernegatively supercoiled’ 336 bp was generated in an identical manner as ‘relaxed’, except for the addition of ethidium bromide (6.5 μg/ml) to the ligation reaction. Ligations were subsequently extracted with butanol (to both reduce the volume and to remove the ethidium bromide), extracted with chloroform, then precipitated with ethanol. The nicked, linearized, and supercoiled minicircle samples were also subjected to butanol and chloroform extraction, and ethanol precipitation in a similar manner to both remove the enzymes and to ensure that any differences observed could not be attributed to differences in how the samples were made. Following ethanol precipitation, DNA was resuspended in 50 mM Tris-Cl pH 8.0, 150 mM NaCl, and 10 mM CaCl_2_. DNA samples were subsequently subjected to multiple rounds of buffer exchange in the same buffer using an Amicon 0.5 ml centrifugal filter to ensure that buffer conditions were equal across all samples. DNA concentrations were determined using a Nanodrop spectrophotometer.

### Geometry and topology of DNA minicircles

DNA supercoiling is defined by the linking number (*Lk*), the total number of times the two single DNA strands coil about one another (24). *Lk* is, thus, an integer number by construction if both strands are covalently closed. If one or both of the strands is not covalently closed, e.g., for nicked and linear DNA, *Lk* can adopt non-integer values. Another quantity determining the shape of DNA is the equilibrium helical repeat *h* defined as the number of base pairs between two locations where the backbones are aligned and is measured in base pairs per turn. The value of *h* is buffer dependent and is ∼10.42 bp/turn in 10 mM CaCl_2_ (13). Using *h*, we can calculate the angle between terminal base pairs of a straight linear DNA segment of a given length *L*, which gives us the reference value *Lk*_0_ = *L*/*h*. Because *h* can, in principle, take any value, *Lk*_0_ is not restricted to integer values and usually has a fractional part. For relaxed 336 bp minicircles, we get *Lk*_0_ = 32.2 while for 672 bp *Lk*_0_ = 64.4. Therefore, the deviation from the most relaxed DNA structure is measured by the difference between *Lk*_0_ and *Lk* denoted by Δ*Lk* = *Lk* − *Lk*_0_, which for the relaxed configuration of the 336 bp minicircle yields Δ*Lk* = −0.2. Δ*Lk* is typically scaled to the DNA length to give the superhelical density σ = Δ*Lk*/*Lk*_0_.

Because *Lk* is constrained to integer values, it is sufficient to report Δ*Lk* rounded to the nearest integer to uniquely identify experimental configurations, as used in Ref. (13). For the relaxed 336 bp minicircle, we round −0.2 to 0. For simplicity, we follow this convention when reporting experimental values in this work. At the same time, we keep track of the fractional parts to accurately compute the elastic properties of the minicircles.

### Gel electrophoresis

DNA samples were analyzed by electrophoresis through 5 % (for 336 bp and 672 bp samples) or 4 % (for 672 bp samples) polyacrylamide gels (acrylamide:bis-acrylamide = 29:1) in Tris-acetate buffer (pH 8.2) containing either 150 mM NaCl and 10 mM CaCl_2_ (5 % gels) or 10 mM CaCl_2_ (4 % gels) at 125 V (∼6 V/cm) for 8 hours. Buffer was continuously recirculated during electrophoresis. DNA samples were also analyzed by electrophoresis through 1.5 % and 3 % agarose gels (Seakem LE agarose, Lonza, Rockland, ME) in TAE (Tris-acetate + 1 mM EDTA) buffer at 100 V for 3 hours. Gels were subsequently stained with SYBR Gold (ThermoFisher Scientific, Waltham, MA), then visualized using a FOTO/ANALYST Investigator imaging system (Fotodyne, Hartland, WI, USA) with quantitation using ImageQuant TL, version 8.1 (GE Healthcare Life Sciences, Marlborough, MA, USA).

### Analytical ultracentrifugation

Linearized, nicked, relaxed, supercoiled, and hypernegatively supercoiled 336 bp minicircles, and supercoiled and nicked 672 bp samples, were measured by sedimentation velocity using an AN50Ti rotor in a Beckman Coulter Optima AUC at the Canadian Center for Hydrodynamics at the University of Lethbridge in Alberta, Canada. For details of minicircles used, see Supplementary Table S1.

All samples were measured in 50 mM Tris-Cl pH 8.0, 150 mM NaCl, and 10 mM CaCl_2_. 460 μl of each sample at an absorbance (A) of 0.6 at 260 nm were loaded into cells fitted with sapphire windows and 12 mm double channel epon charcoal centerpieces (Beckman Coulter, Indianapolis, IN, USA). Data were collected in intensity mode at 260 nm, and at 20 ℃ at five different rotor speeds of 10, 14, 25, 35, and 45 krpm. After data collection at each speed was completed, AUC cells were thoroughly shaken to redistribute the minicircle DNA uniformly. Depending on minicircle topology and length, at 10 krpm, pelleting occurred between 559–770 scans, requiring 50–70 hours. At 45 krpm, pelleting occurred after 88–159 scans, requiring 2–4 hours. The density and viscosity of the buffer, estimated with UltraScan, was 1.00682 g/ml and 1.02667 cP, respectively.

### AUC data analysis

All data were analyzed with UltraScan-III, version 4.0 (6345) (22), using the UltraScan data acquisition module (25). UltraScan fits experimental data to finite element solutions of the Lamm equation, deriving distributions for sedimentation and diffusion coefficients (26,27). Optimization is achieved by parallel distributed data analysis, which was performed on the UltraScan Science Gateway using XSEDE resources (Expanse, Bridges 2, Stampede), and high-performance computing clusters at the University of Montana and University of Lethbridge. The optimization process proceeds through a series of model refinement steps, which employs the two-dimensional spectrum analysis (2DSA) (28). This refinement process removes systematic noise contributions contained in the raw data and obtains exact boundary conditions (the radial positions at the meniscus and the bottom of the cells) as described in (29). The final 2DSA refinement result is used to initialize a genetic algorithm analysis (GA) (30), which is followed by a Monte Carlo GA analysis (31). The total concentration determined from each speed between identical samples was also compared to ensure no material was lost due to aggregation or degradation, and samples were comparable across all speeds for a global analysis. The Monte Carlo GA results from identical samples and different speeds were combined to initialize a global GA analysis over all speeds. UltraScan supports simultaneous fitting to datasets from multiple experiments performed at different speeds. A global analysis benefits from the enhanced signal of the diffusion coefficient at low speeds and the improved sedimentation signal at higher speeds (32,33). This feature also enhances signal-to-noise ratios and improves the confidence limits for the determined hydrodynamic parameters. The global fitting algorithm in UltraScan is further explained in (22).

### Hydrodynamic properties

#### Translational diffusion coefficient

The translational diffusion coefficient *D* is inversely proportional to the translational frictional coefficient *f*,


(1) $$\begin{equation*} D = \frac{k_B T}{f}, \end{equation*}$$

where *k*_*B*_ is the Boltzmann constant, and *T* the absolute temperature. In this work, diffusion occurs at very low concentrations of solute, which allows the analysis of transport coefficients in terms of single-particle properties only. For microscopic solid spheres of radius *R* suspended in a liquid of temperature *T* and viscosity $\eta$ the Stokes-Einstein relationship reads


(2) $$\begin{equation*} D = \frac{k_B T}{6\pi \eta R}. \end{equation*}$$

This relationship can be generalized to non-spherical molecules by introducing the effective hydrodynamic radius *R*_*h*_, defined as


(3) $$\begin{equation*} R_h = \frac{k_B T}{6\pi \eta D}. \end{equation*}$$

In aqueous solvents, macromolecules are typically hydrated, which adds to their apparent size and friction. The value of *R*_*h*_ derived from measured *D* includes these effects.

The hydrodynamic anisotropy of the DNA minicircles was characterized by the frictional ratio *f*/*f*_0_, being the ratio of the measured frictional coefficient *f* and the frictional coefficient *f*_0_ of a spherical particle of the same volume. The anisotropy equals 1.0 for a spherical molecule and exceeds 1.0 for non-spherical molecular shapes.

#### Sedimentation coefficient

The sedimentation coefficient *s* depends on the molar mass *M*, the translational frictional coefficient *f*, and the buoyancy of the particle, which is a function of its PSV, $\bar{v}$, and solvent density ρ,


(4) $$\begin{equation*} s \, =\, \frac{M(1-\bar{v}\rho )}{Nf}, \end{equation*}$$

where *N* is Avogadro’s number. The Svedberg equation describes the ratio of the two parameters measured in a sedimentation velocity experiment, *s* and *D*, and provides a way to estimate the molar mass *M*, if the PSV is known:


(5) $$\begin{equation*} \frac{s}{D} \, =\, \frac{ M(1-\bar{v}\rho )}{N k_B T}. \end{equation*}$$

#### Apparent PSV

Eq. (5) considers a two-component system—an analyte with anhydrous molar mass *M* and a solvent with density ρ. However, our experimental solution also contains buffer components and ions that may be bound to the analytes. The degree of counterions bound to the analyte is dependent on solvent conditions and the ionic strength of the solvent, particularly for charged molecules (34). PSV is defined as the change in volume when one gram of analyte is added to the solvent, and is typically reported in units of ml/g. Because we do not know the precise amount of counterions bound to the analyte, we consider an apparent partial specific volume $\bar{v}^{\prime }$, which can only be considered constant for a single solvent at a constant temperature and pressure. Rearranging the Svedberg equation allows the determination of the apparent PSV, provided the molar mass and the solvent density are known and the sedimentation and diffusion coefficients have been determined experimentally from a sedimentation velocity experiment


(6) $$\begin{equation*} \bar{v}^{\prime } = \frac{1}{\rho }\left( 1 - \frac{Nsk_BT}{MD}\right). \end{equation*}$$

In our case, the molar masses are 207.576 kDa for the 336 bp minicircle and 415.152 kDa for the 672 bp minicircle, as calculated from the sequence (12) using molbiotools.com/dnacalculator. UltraScan automatically estimates the solvent density and viscosity from the buffer composition, and adjusts the experimental *s*_*T*, *B*_ and *D*_*T*, *B*_ values to standard conditions (water at 20 ℃) using the density and viscosity estimates from the buffer components, see ((35), p. 117)


(7) $$\begin{equation*} s_{20,w} = s_{T,B} \frac{(1-\bar{v}^{\prime }\rho )_{20,w}\ }{(1-\bar{v}^{\prime }\rho )_{T,B}} \frac{\eta _{\, T,B}}{\eta _{\, 20,w}}, \end{equation*}$$

(8) $$\begin{equation*} D_{20,w} = D_{T,B}\frac{T_{20}}{T}\frac{\eta _{\, T,B}}{\eta _{\, 20,w}}, \end{equation*}$$

where *s*_*T*, *B*_ is the observed sedimentation coefficient at experimental conditions (temperature *T* = 293.15 K and buffer *B*). However, for the $s_{20,\,w}$ corrections, the partial specific volume of DNA at standard conditions is required, but it is not known to us and impossible for us to measure. While literature values are reported for NaDNA (0.54–0.55 ml/g) (36,37), topoisomers here were studied in 10 mM calcium, which has a higher binding affinity to DNA than Na (38). Hence, we report here the experimentally measured values of *s* and *D* for all topoisomers, and the apparent partial specific volume under experimental conditions, $\bar{v}^{\prime }$, calculated by Eq. (6) and assuming a two component system.

### Finding equilibrium shapes of loops

To model the shapes of DNA minicircles with a given *Lk*, a variant of the Kirchhoff beam theory for inextensible rods (39) was used, which describes the twisting and bending of a uniform elastic filament of constant steric thickness *d*_*s*_, which was set to 20 Å in all computations. The helical repeat of the DNA yields a reference value of *Lk*_0_ = *L*/*h* for a given length *L*.

To model a DNA minicircle with a given *Lk* (and Δ*Lk*), we used an elastic beam representation in which a (closed) beam is characterized by two constants: bending rigidity *A* and geometric torsional stiffness ω (describing the cross-sectional shape, equal to 2/3 for circular cross sections). The energy density has quadratic contributions from the residual excess twist density Ω and local curvature $\kappa$. The total energy is thus given by


(9) $$\begin{equation*} E_\text{rod} = \frac{1}{2}\int A\left(\kappa ^2 + \omega \Omega ^2\right)\mathrm{d}s, \end{equation*}$$

where Ω is computed from *Lk* and the shape of the filament centerline with the help of the Călugăreanu theorem (40)


(10) $$\begin{equation*} Lk\, \, =\, \, Tw + Wr, \end{equation*}$$

where twist (*Tw*) is defined as


(11) $$\begin{equation*} Tw\, =\, Lk_0 \, +\, \frac{1}{2\pi }\int _0^L \Omega \, \mathrm{d}s, \end{equation*}$$

and writhe (*Wr*) is defined in the standard way (41). Scaling by *k*_*B*_*T*, the energy function can be made dimensionless, leaving the (width-to-length) aspect ratio *d*_*s*_/*L* and ω as the only parameters of the model. This approach was used by Coleman and Swigon (42) to categorize equilibrium shapes of looped filaments for a single aspect ratio *d*_*s*_/*L* = 8.2 × 10^−3^ (corresponding to a DNA minicircle of length 718 bp and *d*_*s*_ = 20 Å), which constitutes a benchmark for our computations. Coleman and Swigon began by solving the problem of a free beam segment subject to boundary conditions at each end, and two beams in contact along a contact line. Such solution fragments can be glued together at contact points to form a looped solution, subject to gluing conditions that ensure the continuity of the first two derivatives and appropriate jump conditions to account for beam-beam steric forces. This approach uses the same expression for the beam energy but addresses the energy minimization in a different way—either by solving an ordinary differential equation subject to appropriate boundary conditions when no contact forces are needed or by direct minimization subject to no-overlap constraint when contact forces are present.

### Determination of critical Δ*Lk*

The stability of a computed minicircle shape depends on its Δ*Lk* (42,43). For sufficiently small |Δ*Lk*|, a flat circular configuration is the only equilibrium solution. Upon increasing |Δ*Lk*|, at a thickness-dependent threshold value of critical (minimal) *Lk*_crit_, a figure-8 solution becomes admissible and the flat circular and figure-8 shapes coexist. Supercoiling further, above the thickness-independent threshold of $Lk_\text{max}\, =\, \sqrt{3}/\omega$, the flat circular shape is no longer a solution and only writhed configurations exist (43). For the prediction of *Lk*_crit_ for initial writhing of a minicircle, an approach based on solving an ordinary differential equation for centerline shape was used. The minimal value of |Δ*Lk*| required for writhing can be characterized by the existence of a configuration with a single contact point but with zero contact force. An ordinary differential equation was written for the beam centerline with minimum energy in a Cartesian parametrization subject to the boundary condition of a single contact point and no contact force and solved numerically using Mathematica, where boundary value problems are solved by the shooting method with conjugate gradient descent. For any given separation of the centerline at the self-contact location, one value of residual twist density was found. The relationship between the two was used to derive *Lk*_crit_(*d*_*s*_/*L*).

### Energy minimization of DNA minicircle shapes

Having determined the range of |Δ*Lk*| for which writhed configurations can be stable, the space of admissible configurations was examined and those configurations that minimized the elastic energy were investigated. Here, because of the presence of contact forces, a different numerical method was used. Representing the centerline shape with periodic cubic splines, direct energy minimization was performed over all possible shapes without self-intersections. Curves with 16 nodal points with enforced dihedral symmetry were subjected to a Monte Carlo minimization procedure. The bending energy was calculated directly from curvature using the adaptive Simpson’s algorithm and taking advantage of the twice continuously-differentiable nature of the cubic splines. The precise estimation of *Wr*, required to compute *Tw*, was performed by approximating the curve by 200 linear segments and using an algorithm proposed by Levitt (44) to deal with the singularities of the Gauss formulation. Steric interaction was introduced by tracking self-intersections through a large number (20 *L*/*d*_*s*_) of sample points along the curve and a suitable steric energy penalty. Length constraint was imposed by computing the apparent length at each optimization step using the adaptive Simpson’s method and by imposing an energy penalty for the deviation from the prescribed length. Multiple sets of different control parameters for numerical optimization were tested to ensure both fast convergence and satisfactory precision. All final computations were done with identical discretization and penalty characteristics. For final values of penalty parameters, Monte Carlo procedure parameters, and initial conditions, see data availability section.

### Models for hydrodynamic radius

Solutions for Stokes flow around a slender toroidal object were developed by Johnson (45) that provide an asymptotic approximation in terms of slender-body theory. A fully analytical approach based on toroidal harmonics used by Goren and O’Neill (46) allows exact computations of all elements of the mobility matrix for a torus with an arbitrary aspect ratio. For a rigid, axially symmetric particle of a given length *L* and hydrodynamic thickness *d*_*h*_, the mobility coefficients for translation along the symmetry axis and perpendicularly to it, μ_z_(*L*, *d*_*h*_) and μ_*x*_(*L*, *d*_*h*_), respectively, are sufficient to compute the hydrodynamic radius *R*_*h*_ by taking the inverse of the arithmetic mean


(12) $$\begin{equation*} R_h = \frac{1}{6\pi \eta }\left(\frac{3}{2\mu _x(L,d_h) + \mu _z(L,d_h)}\right). \end{equation*}$$

To theoretically determine *R*_*h*_ for an arbitrarily shaped molecule, a rigid bead model of its structure was constructed and its hydrodynamic radius was calculated using the ZENO software package (47,48). In our case, the configuration of a minicircle was represented by 400 spherical and overlapping beads placed on the shape centerline, with diameters corresponding to the hydrodynamic thickness of the DNA molecule and the distance between overlapping beads summing up to the length of the molecule. This structure was then used to evaluate *R*_*h*_ for the composite particle. The diffusion coefficient at a given *T* and $\eta$ is calculated from the definition of *R*_*h*_ in Eq. (2). The sedimentation coefficient is obtained from the Svedberg relation, eq. (5).

## RESULTS

### Rationale

The study of DNA supercoiling and curvature has benefited from multiple complementary theoretical and experimental approaches. The combination of multiple approaches takes advantage of the knowledge gained from each approach while helping shore up their individual limitations.

We previously (13) used 336 and 672 bp minicircles to study the effect of supercoiling and looping on DNA structure and have extensive 3-D structural data. AUC requires more material than cryoET (13), AFM (19), or other biochemical and biophysical analyses (12). A wide range of supercoiling was explored by testing relaxed, nicked, supercoiled, and hyper-negatively supercoiled minicircle samples. Supercoiled minicircle DNA was obtained from bacterial cells and, therefore, approximates the steady-state level of supercoiling in bacteria. Hypernegatively supercoiled samples have been further manipulated to increase the level of supercoiling and allowed us to determine whether AUC can distinguish different topoisomers from each other. To determine the effect of circularity, linearized DNA samples were also analyzed.

We characterized the minicircle samples by gel electrophoresis, which allows the topoisomer distribution of each sample to be precisely determined. Polyacrylamide gel electrophoresis effectively separates minicircle topoisomers and provides some insight into the conformational differences, although the theory underlying the differential migration is not fully understood.

We then applied advanced theoretical modeling to see whether it can explain the previously observed 3-D conformations of these minicircles (13). It was reasonably successful, and these advanced mathematical models could then be used to help analyze and interpret AUC data. Algorithms used elsewhere to interpret AUC data approximate shape as a sphere to a line, but because of the diversity of shapes of DNA, in this paper a theoretical model was constructed to determine specific shapes and use them to evaluate diffusion and sedimentation coefficients.

### Electrophoretic characterization of DNA minicircles

DNA minicircles were analyzed by polyacrylamide gel electrophoresis. Both the helical repeat and conformation of DNA are sensitive to solution conditions (49,50). Here we used the same conditions used in analytical ultracentrifugation experiments (150 mM NaCl and 10 mM CaCl_2_).

**Figure 1.**
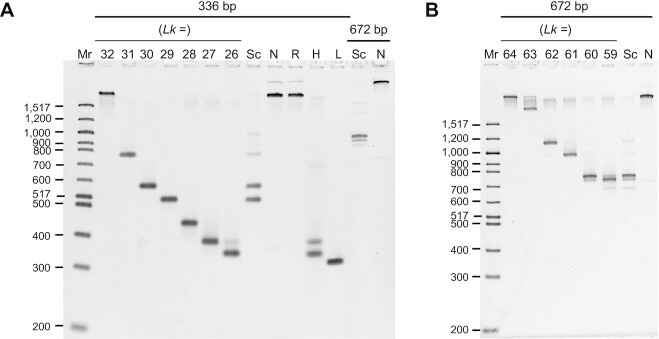
Electrophoretic mobility of minicircle DNA. (**A**) DNA samples were analyzed by polyacrylamide gel electrophoresis (5 % polyacrylamide) in 150 mM NaCl and 10 mM CaCl_2_ (the same conditions used in analytical ultracentrifugation). Mr: 100 bp DNA ladder, lanes 2–8: 336 bp minicircle topoisomer markers (*Lk* as indicated), lanes 9–13: 336 bp minicircle DNA samples (Sc: ‘supercoiled,’ N: nicked, R: relaxed, H: ‘hypernegatively supercoiled,’ L: linear), lanes 14–15: 672 bp DNA samples (Sc: ‘supercoiled,’ N: nicked). (**B**) Determination of topoisomer identity in 672 bp samples. DNA samples were analyzed by electrophoresis on a 4 % polyacrylamide gel in the presence of 10 mM CaCl_2_. Mr: 100 bp DNA ladder, lanes 2–8: 672 bp minicircle topoisomer markers (*Lk* as indicated), lanes 9–10: 672 bp DNA samples (Sc: ‘supercoiled’ as isolated from the bacteria, N: nicked).

Supercoiled topoisomers migrated much more rapidly on the polyacrylamide gel than relaxed topoisomers (Figure 1). In comparison, the different topologies had relatively similar mobilities on an agarose gel (Supplementary Figure S1). This difference in migration on polyacrylamide gels can be at least partially explained by the relative compactness of supercoiled minicircle conformations (13). The nicked and relaxed topoisomers had near-identical migration, suggesting that the single-strand break in the nicked minicircle does not significantly affect the global conformation. The lack of difference is explained by the number of helical turns in the 336 bp minicircle studied being close to a perfect integer value of 32 (under these conditions), resulting in the base pairs flanking the nick site being in close rotational alignment, allowing for favorable base stacking across the nick (12). We previously showed that when the rotational alignment is out of phase (i.e., when the number of helical turns deviates from a perfect integer value), the effect of a nick on polyacrylamide gel migration is much more pronounced (12).

The topoisomer distribution for the samples taken through to AUC analyzes was measured from quantification of digital images of the fluorescently stained gels using image analysis software. The ‘supercoiled’ 336 bp sample contained primarily Δ*Lk* = −3 (48 %), Δ*Lk* = −2 (41 %), and Δ*Lk* = −1 (7 %) topoisomers. The sample also contained trace amounts of nicked 336 bp (1 %) and supercoiled 672 bp (3 %) minicircle DNA. The topoisomer distribution obtained (mean σ ∼ −0.08) reflects the supercoiling level in the bacterial strain used to generate the minicircles. The ‘hypernegatively supercoiled’ sample contained primarily Δ*Lk* = −6 (61 %) and Δ*Lk* = −5 (33 %) topoisomers, with trace amounts of nicked 336 bp (4 %) and supercoiled 672 bp (3 %) minicircle DNA. This sample (with mean σ ∼ −0.15) is representative of the very high levels of dynamic supercoiling generated transiently during transcription.

The supercoiled 672 bp sample contained primarily Δ*Lk* = −4 (63 %), Δ*Lk* = −5 (24 %), Δ*Lk* = −6 (6 %) topoisomers, and trace amounts of nicked 672 bp (2 %), Δ*Lk* = −2 (4 %) and Δ*Lk* = −3 (2 %) topoisomers. The identity of the topoisomers present in the supercoiled 672 bp sample was determined on a separate gel with 672 bp topoisomer markers. The topoisomer distributions of each sample are compiled in Supplementary Table S1.

### Analytical ultracentrifugation of DNA minicircles

Apparent PSVs obtained from a global multispeed genetic algorithm-Monte Carlo analysis for each minicircle sample are summarized in Table 1. The derived PSV values did not show any apparent pattern that would indicate a dependence of the PSV on topoisomer conformation, and resulted in a near constant value of 0.482 ±  0.011 ml/g over all tested minicircles (see Table 1). The average PSV for 336 bp minicircles was 0.477 ml/g while the average PSV for 672 bp minicircles was 0.494 ml/g.

**Table 1.** Apparent partial specific volume for DNA minicircle topoisomers in the buffer

Partial specific volume^a^ in mg/l		
Sample	336 bp	672 bp
Linear	0.479	ND
Relaxed	0.470	ND
Nicked	0.469	0.495
Supercoiled	0.488	0.494
Hypernegatively supercoiled	0.479	ND
Average value^b^	0.482 ± 0.011	

^a^Determined by global sedimentation velocity analysis using the known molar masses. ^b^For all the 336 bp and 672 bp minicircle DNA species. ND, not determined.

**Figure 2.**
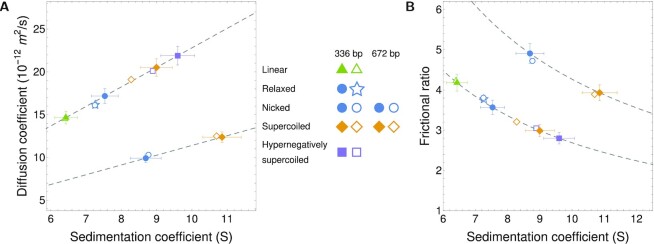
Measured and predicted diffusion and sedimentation coefficients for DNA minicircles. AUC measurements using global Monte Carlo-Genetic Algorithm analysis are marked as empty symbols. Theoretical predictions are presented with filled symbols. (**A**) Diffusion coefficient as a function of the sedimentation coefficient for topoisomers of 336 bp (upper branch) and 672 bp (lower branch) minicircle DNA. Dashed lines represent the constant mean value of PSV determined from AUC experiments upon which theoretical predictions of the sedimentation coefficient are based. Experimental and theoretical data for 336 bp and 672 bp relaxed and nicked minicircles overlay almost completely and thus are impossible to discern in the plots. (**B**) Frictional ratio as a function of the sedimentation coefficient. Sedimentation coefficients *s* are measured in svedberg units (S), with 1 S = 10^−13^ s.

Using the determined average PSV value of 0.482 ml/g, a frictional ratio *f*/*f*_0_ was derived from the sedimentation and diffusion coefficients obtained in the global analysis. Plots of the frictional ratio as a function of sedimentation coefficient and the diffusion coefficient as a function of the sedimentation coefficient are shown in Figure 2. DNA topology had a significant effect on sedimentation and diffusion coefficients. In contrast to polyacrylamide gel electrophoresis, for which linear migrates fastest (Figure 1), circular molecules (nicked, relaxed, supercoiled, and hypernegatively supercoiled) all sedimented faster than linear.

AUC was additionally able to differentiate relaxed, supercoiled, and hypernegatively supercoiled samples. The supercoiled or hypernegatively supercoiled samples containing a mixture of topoisomers behaved as single species in AUC. This result can be rationalized by noting that the topoisomers in experiments differ in *Lk* by 1 only. While the difference between nicked and supercoiled minicircles is clearly seen, it may be difficult to resolve by AUC minicircle topoisomers differing by only one or a few *Lk*.

### Predicted shapes of DNA minicircles

Before employing our mathematical models to predict the effect of supercoiling and curvature on the hydrodynamic properties of DNA, we needed to first test how well these models predicted known equilibrium 3-D minicircle shapes. We adopted the strategy of building a coarse-grained representation of the equilibrium shapes of the minicircles obtained using our energy minimization codes. These models are reduced representations of macromolecules still capable of retaining key physical aspects (51). This approach has been highly successful in calculations of biomolecule properties in solution (52). Having found the shapes of DNA minicircles, we calculated the hydrodynamic radius for each conformation. The hydrodynamic radius was used to calculate the sedimentation and diffusion coefficients. We also tested our models against the 3-D structures of these minicircles, previously observed experimentally (13). Our aim was to develop a practical predictive theoretical framework to determine the measured transport coefficients.

#### Electrostatic screening and Brownian contributions

DNA molecules have substantial charge, which can be exploited, e.g., in electrophoretic measurements of DNA of different lengths. A qualitative comparison of the elastic and electrostatic forces is possible by considering scaling arguments. The Debye-Hückel equation is a well-established model of electrostatic interaction in a buffer containing counterions (53). In this approach, the interaction potential decays exponentially with separation due to screening. The decay rate is quantified by a characteristic distance, the Debye length (*R*_*D*_). Comparing *R*_*D*_ with the typical distances between base pairs gives a crude estimation of the influence of electrostatic forces. We estimated *R*_*D*_ for our setup to be 1.45 Å from the ionic strength of 230 mM using an ionic strength-based estimate $R_D^{-1}\, =\, \kappa _0 \sqrt{C_s}$, where *C*_*s*_ is the molar salt concentration in moles per liter and $\kappa_0$ = 0.329 Å^−1^l^1/2^mol^−1/2^, as reported by Lim *et al.* (54). Thus the Debye length is much smaller than an average distance between different segments of the DNA molecule. Notably, this value of *R*_*D*_ was also much lower than that of earlier work, such as 30 Å in Ref. (55) for different buffer conditions.

The persistence length *P* of polyelectrolytes is the sum of two contributions,


(13) $$\begin{equation*} P\, =\, P_\text{in}\, +\, P_\text{el}, \end{equation*}$$

where *P*_in_ is an intrinsic persistence length due to the rigidity of the backbone, and *P*_el_ is an electrostatic persistence length, which accounts for buffer-dependent repulsion between neighboring ionic sites (56). The latter can be related to the Debye length as *P*_el_ = *R_D_*^2^(4*l*_*B*_)^−1^, with *l*_*B*_ being the Bjerrum length, according to the Odijk-Skolnick-Fixman theory (57–59). For linear or relaxed double-stranded DNA at room temperature, the persistence length in 0.1 M NaCl is approximately 500 Å (150 bp) (60). Based on persistence length, one can define effective bending and twisting energies for a circular shape as


(14) $$\begin{equation*} E_\text{bend} = \, 4\pi ^ 2 k_B T \frac{P}{L}=\, 4\pi ^2 \frac{A_\text{eff}}{L}\quad \text{and} \end{equation*}$$

(15) $$\begin{equation*} E_\text{twist} =\, 6\pi ^2 k_B T (\Delta Lk)^2 \frac{P}{L}=\, 6\pi ^2 (\Delta Lk)^2 \frac{A_\text{eff}}{L}, \end{equation*}$$

which, via eq. (13), include both elastic and electrostatic contributions. Here, *A*_eff_  =  *k*_*B*_*TP* is the effective bending rigidity of the DNA. The remaining long-ranged electrostatic contribution *E*_lr_ can be estimated as the interaction energy between *N* equal charges *q* at a typical distance comparable to the radius of the loop, $L / 2 \pi$, which amounts to


(16) $$\begin{equation*} E_\text{lr} \approx \frac{q^2 N}{2\epsilon L}\exp \left(-\frac{L}{2\pi R_D} \right), \end{equation*}$$

where ε is the dielectric permittivity of the buffer. Note that these estimates do not take into account short-range electrostatic interactions between distant parts of the DNA that may come close together when supercoiled. For a circular loop with |Δ*Lk*| = 2 and equivalent length of 336 bp, we found *E*_bend_ ≈ 17 *k*_*B*_*T*, *E*_twist_ ≈ 105 *k*_*B*_*T*, and a negligible value of *E*_lr_. The effective bending and twisting energies considerably exceed the typical energy of thermal fluctuations, *k*_*B*_*T*, which is somewhat surprising given that the minicircles are longer than *P*. This result may be a consequence of the relatively high energy stored in the form of bending energy *E*_bend_, which renders the circular configuration less prone to Brownian shape disturbances than a torsional-stress-free linear DNA. We therefore use the stiff beam approximation to describe minicircle shapes.

#### Critical ΔLk for writhing

The shape of a DNA minicircle depends primarily on |Δ*Lk*| and the width to length ratio *d*_*s*_/*L*. To predict 336 bp and 672 bp minicircle conformations for Δ*Lk*  =  0 to |Δ*Lk*|  =  5, we developed a theoretical framework that assumed that DNA can be modeled by a continuous elastic beam of Δ*Lk* with the steric thickness *d*_*s*_ and stiffness *A* as determined by the inter-phosphate distance and persistence length, respectively. The previous theoretical study of Coleman and Swigon (42) focused on a circularized 718 bp DNA fragment and determined the structure of possible stable configurations, describing the contact diagrams in great detail. Our results were consistent with their findings for a particular thickness to length ratio (*d*_*s*_/*L* = 0.0082) but were applied to an arbitrary aspect ratio.

**Figure 3.**
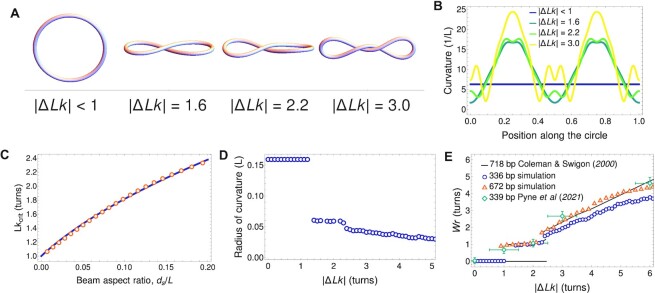
Elastic equilibrium shapes of model DNA minicircles. (**A**) Energy minimizing shapes of 336 bp minicircle of various Δ*Lk* (with *d*_*s*_/*L* = 0.018). For small |Δ*Lk*| values (<1.6), loops adopt open circle configurations. For intermediate values (Δ*Lk* = 1.6 or 2.2), a single point of polymer contact is observed; for larger values of Δ*Lk* (>2.2), continuous contact is observed. (**B**) Curvature distribution along the supercoiled loop centerline in shapes corresponding to panel (**A**). The position along the loop is measured from the point of contact (or the center of symmetry in multiply touching configurations). **(C)** *Lk*_crit_ above which a writhed configuration can be stable, plotted as a function of the beam aspect ratio (steric diameter to length, *d*_*s*_/*L*). Solid line shows the approximation of eq. (17). **(D)** Minimal radius of curvature along the loop as a function of Δ*Lk* for the 336 bp minicircle. This radius decreases monotonically with Δ*Lk*, leading to increasing bending stresses. **(E)** Writhe of energy-minimizing shapes as a function of the aspect ratios *d*_*s*_/*L* = 0.018, 0.0090, and 0.0082 for 336 bp, 672 bp, and 718 bp DNA minicircles respectively. For small values of |Δ*Lk*|, only flat (open circle) configurations are permitted but above *Lk*_crit_, one or more twists is relaxed by writhing. For large values of |Δ*Lk*|, around 90 % of torsional energy is relaxed by shape deformation. Our results are shown next to the continuum model predictions of Coleman and Swigon (42) and atomistic MD simulations of Pyne *et al.* (19).

In Figure 3A we present the shapes obtained by our minimization procedure. Close to Δ*Lk* = 0, circular configurations were stable. At a critical value of |Δ*Lk*|, a figure-8 shape became energetically favorable, and the loop writhed to relax excess twist. For higher |Δ*Lk*|, the number of self-contacts increased, leading to a more writhed configuration. In this process, regions of high and low curvature emerged along the loop, as sketched in Figure 3B, with higher curvature present for higher values of |Δ*Lk*|, as expected intuitively. For open configurations, the curvature was nearly constant and equal to $2 \pi / L$. The position along the loop changed from 0 to 1 and was measured from the point of contact or (in the case of multiple contacts) from the center of symmetry. With increasing |Δ*Lk|*, regions of increased curvature appear far away from self-contact points.

For larger |Δ*Lk*|, increased curvature was present even at points close to the contact line. The variation of curvature along the loop centerline, shown in Figure 3C, might hint at sites of potential base pair instability or other configurational changes. The effect of localized curvature regions would be further compounded by the transmission of mechanical stress along the DNA backbone to promote DNA kinking and base pair disruption at distant sites (13,19,61,62) as reported by Fogg *et al.* (12).

The value of *Lk*_crit_ for the open circle-figure-8 transition depends on the aspect ratio *d*_*s*_/*L*. In Figure 3C, we show the dependence of *Lk*_crit_ on the aspect ratio numerically, showing that for thicker beams a transition to writhed configurations required an increased negative supercoiling. A very thin filament can be stably writhed for almost any value of |Δ*Lk*| greater than 1. However, for beams with a larger thickness, more torsional stress was required to stabilize writhed configurations and prevent them from unwrithing to a circular, open configuration.

For increased values of *d*_*s*_/*L*, we also empirically found a convenient approximate expression for


(17) $$\begin{equation*} Lk_\text{crit} = \sqrt{1+ 23.1 \frac{d_s}{L}}, \end{equation*}$$

which we show in Figure 3C. To further characterize the writhed shapes, in Figure 3D we plotted the minimal radius of curvature of a twisted beam as a function of |Δ*Lk*|. For low values of |Δ*Lk*|, the constant value reflected the purely circular equilibrium. Above *Lk*_crit_, the loop became writhed, with an increased curvature at the apices. The monotonic decrease of radius of curvature with |*ΔLk*| showed that increasingly supercoiled beams tended to have tighter bends and, therefore, stored larger bending energy. Finally, in Figure 3E, we present the writhe of the resulting configuration, calculated with our method as a function of |*ΔLk*|. The values were calculated for both lengths–336 and 672 bp. For comparison, we also plotted the results of computations of Coleman and Swigon (42) for *L* = 718 bp as a solid line, and results of base pair-resolution MD simulations of Pyne *et al.* (19) for a 339 bp minicircle, confirming the observed trend. The total *Wr* as a function of |Δ*Lk*| seemed to be weakly dependent on the minicircle length. Writhing, therefore, emerged as a universal mechanism of stress release for twisted loops.

#### Shape and stability of supercoiled configurations

Although purely elastic considerations suggest a plethora of possible writhed configurations, we mostly observed the simple minicircle conformations in the cryoET measurements of Irobalieva *et al.* (13), rather than the more intricate shapes. The latter have higher energies and are thus less frequently realized (42). For a given Δ*Lk* and at low temperatures, the shapes associated with higher energies were unfavorable compared to the ground state solution determined by our energy minimization procedure.

**Figure 4.**
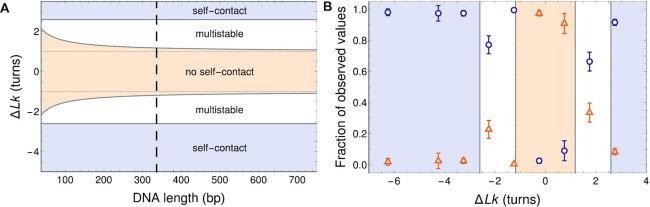
Regimes of shape stability for model DNA minicircles. (**A**) Phase diagram of different regimes of stability of supercoiled DNA as a function of Δ*Lk* and DNA length. The phase space is divided into three regions: only open circular configurations permissible (tan-shaded area), both circular and writhed configurations permissible (multistability; white area), and only writhed configurations permissible (blue-shaded area). The dashed line marks the 336 bp minicircles, for which experimental shape data are available. (**B**) Plot of the relative occurrence rate of configurations with (blue circles) and without (orange triangles) points of self contact, based on cryoET shapes of 336 bp minicircles measured by Irobalieva *et al.* (13) and overlayed on our theoretical stability predictions with the same color coding as in panel (**A**). Error bars represent 2 standard deviations.

Equilibrium shapes measured in cryoET experiments in Ref. (13) were divided into eight groups termed open circle, open figure-8, figure-8, racquet, handcuffs, needle, rod, and other. For a quantitative comparison, we reduced the complexity by distinguishing only between contact-free (corresponding to open minicircle configurations) and self-touching (corresponding to writhed minicircle configurations) solutions. Using the elastic beam framework, we determined the regions of stability of these solutions in terms of Δ*Lk* for a given DNA loop length. We present them in Figure 4A. The orange-shaded region close to Δ*Lk* = 0 favors an open circle as the stable configuration. For under-twisted configurations, we identified a region of multistability, where we found both open circular and figure-8 shapes. Finally, when the inherent Δ*Lk* was large enough, marked by the blue shading in the figure, the self-touching shapes became the only stable energy minimum. The stable circular shape region narrowed down from |Δ*Lk*| < 1.1 for 336 bp minicircles to |Δ*Lk*| < 1.2 for 672 bp and 718 bp minicircles.

For the particular length of *L* = 336 bp, in Figure 4B we compared the theoretical predictions to the cryoET data (13). We measured the fractions of open and self-touching configurations in the population of minicircle shapes. For Δ*Lk* close to zero, we saw that most of the shapes were open loops, with only a fraction of about 10 % showing self-contact. This situation changed with increased Δ*Lk*. In the predicted region of multistability, we saw that the fractions of writhed configurations increased, but there was still a pronounced population of open circular shapes that vanished almost completely when increasing |Δ*Lk*|. This observation is in excellent agreement with our prediction for the new stability region at |Δ*Lk*| > 2.6. In particular, the most relaxed state (closest to Δ*Lk*  =  0) was slightly undertwisted (Δ*Lk* < 0). For configurations with Δ*Lk* = −0.2 and Δ*Lk* = 0.8, only open configurations were predicted while for Δ*Lk* = −2.2, −1.2 and 1.8, we observed large conformational variability corresponding to two solutions in the uniform elasticity model. For Δ*Lk* = −4.2, −3.2, and 2.8, open configurations were no longer permitted by the uniform elasticity theory and these open configurations were largely absent in the prior cryoET measurements for these topoisomers. Interestingly, an outlier at Δ*Lk* = −1.2 was observed in the cryoET data, where we saw a surprising lack of open circular configurations, which could not be simply explained by our coarse-grained model. One potential explanation for this puzzling observation has been postulated that involves a coupling of limited base pair disruption with writhing (12).

### Predictions of hydrodynamic radius of DNA minicircles with different Δ*Lk*

#### Non-writhed configurations

Configurations with small values of Δ*Lk* adopt toroidal conformations; for those configurations, we have found a convenient expression for the hydrodynamic radius, consistent with asymptotic solutions by Johnson and Wu (45) and Johnson (63)


(18) $$\begin{equation*} R_h = \left(\frac{L}{2\pi } + \frac{d_h}{2}\right)\frac{72\pi \left(\frac{1}{16 x^2} + 1\right)x }{25x + 6(11x+8)\log 8x + 16}, \end{equation*}$$

with *x* = *L*/π*d*_*h*_. Here, log  denotes the natural logarithm. Notably, Eq. (18) agrees with the numerical results of Goren and O’Neill (46) even for non-slender tori. For more slender tori, when *L*/*d*_*h*_ > 30, a simpler expression can be fitted without loss of accuracy, given by


(19) $$\begin{equation*} R_h = L\left(\frac{11}{6}\log \left(\frac{2L}{d_h}\right) + 1.13\right)^{-1} \end{equation*}$$

as obtained by Adamczyk *et al.* (64) from the numerical results of the bead model shape approximations. Qualitatively, *R*_*h*_ is of the same order of magnitude as the experimental DNA lengths and thus it approximately scales with the mass of the DNA molecule. This scaling for approximating DNA is in contrast with globular models used for some proteins that scale with the cubic root of their mass instead.

#### Writhed configurations

Configurations with intermediate values of |Δ*Lk*| adopted either toroidal or writhed shapes. To generate hydrodynamic predictions for DNA minicircles with larger values of |Δ*Lk*|, we combined elastic energy minimization with hydrodynamic bead models. To this end, we used equilibrium shapes obtained for each |Δ*Lk*| within the elastic beam model described before, and produced their representation as a collection of 400 rigidly attached and overlapping spherical beads arranged such that the length and thickness of the DNA molecule were left unchanged. The bead model was then used to calculate the hydrodynamic mobility of the conglomerate within the ZENO package (47). We estimated the error of our predictions of the hydrodynamic radius to be about 5 % by comparing the results of bead-model calculations to known analytical solutions for highly symmetric shapes of model molecules.

**Figure 5.**
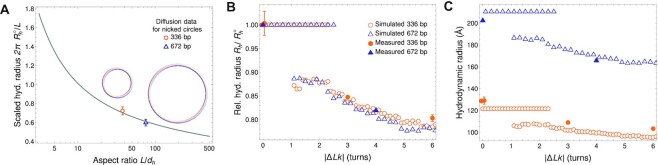
Hydrodynamic radius of DNA minicircles. (**A**) Hydrodynamic (hyd.) radius $R_h^{\circ}$ of open-circular DNA, scaled by the geometric radius *L*/2π of a torus, plotted for a range of DNA aspect ratios *L*/*d*_*h*_. Comparing the ZENO results for toroidal particles (solid line) to diffusion measurements of minicircles with 336 bp and 672 bp yields the fitted (common) hydrodynamic thickness *d*_*h*_ = 29.4 Å. We note that ZENO approximation yields high-precision results for toroidal particles (48). This value was used in all subsequent computations. Circular sketches representing molecules preserve both the relative scale and thickness. Note the logarithmic scale on the horizontal axis. (**B**) *R*_*h*_ of minicircle shapes, relative (rel.) to the hydrodynamic radius $R_h^{\circ}$ of the relaxed, open-circular shape for a range of |Δ*Lk*|. We present results of simulations (open circles and triangles) along with experimental data for 336 bp (filled diamonds) and 672 bp (filled squares) minicircles. For 336 bp (nicked), 336 bp (relaxed), and 672 bp (nicked), the simulations show a region of constant *R_h_* where the shape is independent of |Δ*Lk*|. For supercoiled 336 bp containing a mixture of Δ*Lk* = −1, −2 and −3, the *R_h_* is about 15 % smaller than that of an open circle. For larger values of |Δ*Lk*|, the theoretical approach seems to correctly grasp *R_h_* of the resulting highly compact conformers. **(C)** Absolute values of the *R*_*h*_ for minicircles from (**B**), with the same symbols.

Our modeling approach reduced the problem of finding diffusion and sedimentation coefficients to the computation of the hydrodynamic radius *R*_*h*_. Once calculated theoretically, *R*_*h*_ was used to predict the diffusion coefficient from the Stokes-Einstein relation, Eq. (2), provided that the viscosity of the environment $\eta$ is known. The presented results were adjusted for the buffer viscosity. The prediction of *R*_*h*_ for DNA of any shape required additionally the knowledge of the hydrodynamic thickness of the loop *d*_*h*_. To determine the effective thickness of the molecules (related to the existence of a hydration layer), we assumed that, as a local property, it does not depend on the shape. Then, we used the measured AUC data for nicked and relaxed (Δ*Lk* = 0) 336 bp and 672 bp minicircles, which we assumed to be toroidal. Knowing the dependence of the DNA *R_h_* on the molecule aspect ratio from the ZENO software tool, we compared it to the AUC results for the *R_h_* of the 336 bp and 672 bp DNA minicircles, and we fitted the same value of *d*_*h*_ to both minicircles to reproduce their experimental *R_h_*. The dependence of *R*_*h*_ on the aspect ratio is presented in Figure 5A, together with the two measured values. Fitting the theoretical curve to these two data points yielded the hydrodynamic diameter of *d*_*h*_ = 29.4 Å (corresponding to *d*_*h*_/*L* = 2.6 × 10^−2^ for the 336 bp minicircles and *d*_*h*_/*L* = 1.3 × 10^−2^ for the 672 bp minicircles). We used this value for all subsequent calculations. We note, however, that the estimation of *d*_*h*_ from diffusion measurements cannot be precise due to the logarithmic dependence of hydrodynamic parameters on this value, so the fitted value should be treated as more approximate than the number of digits provided.

The value of excess thickness over inter-phosphate distance was significantly larger than that reported by Fernandes *et al.* (65) (22.8 Å)—without the details of the solvent ionic strength; however, as argued by Penkova *et al.* (66), the hydration shell can be as thick as 16 Å, corresponding to diameters as large as 40 Å. This value is sensitive not only to the ionic strength of the solvent, but also to the details of ion composition (67). Moreover, small deviations from the toroidal shape of the nicked and relaxed DNA shapes are expected, as caused by Brownian motion.

Figure 5B demonstrates that the difference of shapes with different |Δ*Lk|* is significant and similar in theory and experiments. We present therein the hydrodynamic radius for writhed configurations calculated for the two investigated DNA lengths with the same thickness (*d*_*h*_ = 29.4 Å). The plot shows the radius relative to that of a toroidal particle of the same *d*_*h*_, denoted by $R_h^{\circ}$. We normalized theoretical results by calculations at Δ*Lk* = 0, while experimental data are rescaled by respective results for a nicked/relaxed configuration of a minicircle of a given length. Open circles and triangles in the graph mark theoretical results. For the 336 bp minicircle, two experimental values were available, namely that of a nicked and relaxed configuration. In these cases, we rescaled the experimental data by the mean of the two radii. The values of the relative radii are equal to unity for Δ*Lk*  =  0 by definition, but even the non-zero Δ*Lk* simulations predicted a region of stable circular configurations with unchanged *R_h_*.

Increasing |Δ*Lk*| led to the emergence of highly writhed configurations, which tended to be more compact and therefore had a smaller *R_h_* than the open circular configurations seen at low |Δ*Lk*|. We predict that topological writhing could reduce *R*_*h*_ by about 15 % in experimentally relevant conditions as shown in Figure 5B.

We additionally plotted available experimental results for relaxed and supercoiled minicircles, which compared favorably with theoretical predictions. The theory seems to correctly determine *R*_*h*_ obtained from AUC measurements, which opens an efficient route to calculate the hydrodynamic transport coefficients also for highly writhed conformations. In Figure 5C we also presented the same results in absolute terms. Because the fitted value of hydrodynamic thickness in Figure 5A lies between the estimates based on 336 bp and 672 bp only, we see the value of *R*_*h*_ for Δ*Lk*  =  0 to be slightly overestimated by the simulation for 672 bp and underestimated for 336 bp. We emphasize that this difference is an effect of the thickness fitting procedure. This systematic difference can be caused by slightly non-toroidal DNA shapes caused by Brownian motion.

Our AUC data, together with the theoretical modeling shown in Figure 5, yield a length-invariant observation—the ratio of *R*_*h*_ of open circular (nicked or relaxed) minicircle topoisomers to the *R*_*h*_ of compact (supercoiled) topoisomers is approximately 5:4. This value holds true for both 336 bp and 672 bp minicircles, even though the length *L* is longer than the persistence length *P* for 672 bp minicircles, with *L* ≈ 4*P*. This length-invariant ratio of 5:4 holds true as long as thermal effects are negligible. This theoretical result is particularly robust because it is independent of the viscosity of the buffer or the minicircle length. We attributed this robustness to the logarithmic dependence of the hydrodynamic models on the thickness of slender bodies. Additionally, the ratio of *R*_*h*_ of 336 bp linear to *R*_*h*_ of 336 bp nicked is approximately 7:6.

These results can also be viewed in absolute rather than relative terms (Figure 3), where the theoretical predictions for the values of the sedimentation and diffusion coefficients are displayed together with the experimental results. We obtained a good agreement for the larger of the two minicircles (672 bp) and for linear, relaxed, and nicked samples of the smaller minicircle (336 bp). For highly writhed configurations of the smaller minicircle, however, theoretical predictions suggest a more compact conformation than observed. This result could be attributed to the limits of applicability of the linear elasticity theory to the very tight bend radii necessary for energy minimization in these configurations, or to a rough estimate of shapes and width of nicked and relaxed minicircles.

To test the effectiveness of our modeling approach, in Table 2 we compared the measured and predicted values of *D* and *s*. To compute *D*, data from nicked samples were used as a calibration of the only fitting parameter: the effective hydrodynamic diameter of the DNA molecule. With the use of *d*_*h*_, all the *D* values were purely theoretical predictions based on the DNA length and the value determined in separate experiments.

**Table 2.** Comparison of predicted and measured diffusion and sedimentation coefficients in the buffer

		*D* (μm^2^/s)		*s* (S)	
Sample	|Δ*Lk*|	predicted	measured	predicted	measured
336 linear	not applicable	14.7 ± 0.7	14.5	6.4 ± 0.3	6.40
336 relaxed	0	17.2 ± 0.9	16.2	7.5 ± 0.4	7.26
336 nicked	0	17.2 ± 0.9	16.1 ± 0.4	7.5 ± 0.4	7.25 ± 0.02
336 supercoiled^a^	**3**, 2, 1	20.5 ± 1.0	19.0	9.0 ± 0.5	8.28
336 hypernegatively supercoiled^b^	**6**, 5	21.9 ± 1.1	20.1 ± 0.2	9.6 ± 0.5	8.89
672 nicked	0	9.9 ± 0.5	10.3	8.7 ± 0.4	8.77
672 supercoiled^c^	**4**, 5, 6	12.4 ± 0.6	12.5	10.9 ± 0.5	10.72

^a^Predictions done for the dominant species in the mix (Δ*Lk* = −3) highlighted in bold. ^b^Predictions done for the dominant species in the mix (Δ*Lk* = −6). ^c^Predictions done for the dominant species in the mix (Δ*Lk* = − 4). Where experimental errors are not shown, the confidence limits from the global Monte Carlo analysis of the AUC data were exactly 0. We roughly estimated the errors of theoretical values as 5 %. Sedimentation coefficients are measured in svedbergs, with 1 S = 10^−13^ s. Experimental confidence limits are reported only when the last digit of the result would be affected.

**Figure 6.**
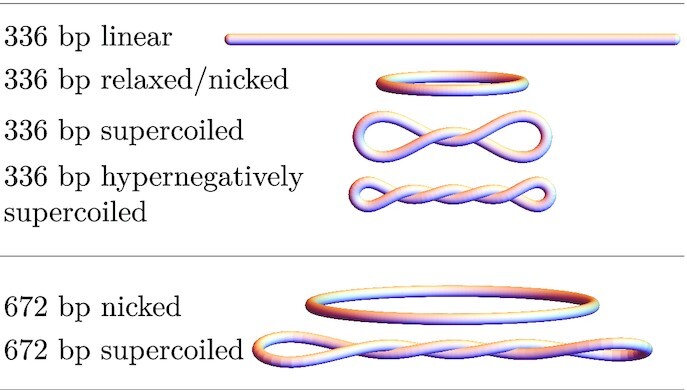
Sketches of model shapes in a given minicircle configuration used for hydrodynamic simulations with Δ*Lk* specified in the caption of Table 2. The sketches have realistic aspect ratios (*d*_*h*_/*L*) and preserve the relative size.

Because supercoiled and hyper-supercoiled samples contain mixtures of minicircles with different Δ*Lk*, theoretical predictions are given for the most common species in a mixture. These configurations are sketched in Figure 6. To calculate *s*, we used the approximately constant value of the PSV determined from the AUC experiments. In all cases, the deviation between theory and experiment was below 8 %. The simplified hydroelastic model provides practical estimates of the experimentally accessible quantities, and thus may be used to discern populations of minicircles differing in Δ*Lk*.

Our modeling experiments reveal that the change of Δ*Lk* by one turn (for example from Δ*Lk* = −4 to Δ*Lk* = −5) changes the frictional ratio by ∼2 %—a value similar to theory-experiment deviations and similar to the noise level coming from the Monte Carlo optimizations of energy minimizing shapes. Such small gradients could be the reason why AUC measurements cannot distinguish topoisomers that differ in Δ*Lk* by only a single turn, especially if the concentrations of the different topoisomers in the mix differ. The samples analyzed by AUC contained a mixture of topoisomers that behaved as a single species because the major species in each sample were within one Δ*Lk* of each other.

## DISCUSSION

Methods that measure the hydrodynamic radius *R*_*h*_, such as gel electrophoresis and diffusion-sedimentation AUC experiments, can be qualitatively described as sorting molecules by their size. Unlike for gel electrophoresis, however, when assessing diffusion via AUC, size contributions can be separated from viscosity, temperature, and PSV by the appropriate scaling, which yields *R*_*h*_.

In electrophoresis, molecules squeeze their way through pores in the gel matrix. One of the major differences between agarose and polyacrylamide is the average pore size, with agarose gels typically having larger sized pores (68). In typical electrophoresis experiments, the primary determinant of the migration speed is the molecular weight and charge of the molecule. Here, the DNA molecule of a given length has a fixed charge and molecular weight, but the speed of migration through the matrix of small pores like the polyacrylamide gel varies greatly when the shape of the molecule is changed (although charge, and thus cations and hydration shell may be altered with negative supercoiling (Randall 2006; Randall 2009)). Comparing gel results to the AUC data allows us to confidently say that knowing the *R*_*h*_ is insufficient to predict its electrophoretic mobility as the gel electrophoresis and AUC separated the minicircle topoisomers differently. Supercoiling influences minicircle electrophoretic mobility much more than hydrodynamic mobility (as measured by AUC); the same change in supercoiling increases electrophoretic mobility by 400 % while increasing mobility only by 20 %. Differences between the methods can be even more dramatic. The linearized 336 bp minicircle showed 600 % increase of electrophoretic mobility, while at the same time (in the AUC measurements) having a 14 % *smaller* hydrodynamic mobility as compared to nicked or relaxed form.

Introducing circularization presents a significant obstacle to extending quantitative electrophoresis methods, such as one proposed by Ziraldo *et al.* (69). Calibrating gel electrophoresis measurements with a ladder of relaxed or nicked circular DNA of different lengths would be insufficient because the contribution of the degree of supercoiling strongly affects the apparent DNA mass derived from that method (at least by a factor of two). These difficulties are further compounded by the dependence on the absolute value of applied electrophoretic-mediated force, as reported by Iubini *et al.* (70). For large electrophoretic-mediated forces, linear DNA is expected to migrate faster than the circular form, which is opposite to what occurred in AUC, while for small forces, the circular form migrates faster than the linear, which is the same as in AUC. One possible explanation for the differences between electrophoresis and AUC is that as the DNA is pulled through the pores of the gel matrix, it has to change its shape (this would be most significant for the Δ*Lk* = 0 samples) whereas there is no sieving in AUC. AUC, therefore, better reflects the solution properties of DNA, while gel electrophoresis offers higher resolution for separation by means we do not yet fully understand.

Diffusion-sedimentation measurements provided by AUC allow accurate hydrodynamic modeling. To elucidate the shapes and properties of DNA minicircles, we proposed a coarse-grained model that represents the DNA as an elongated, uniform elastic beam. By minimizing the elastic energy of a beam with a given superhelical density, we were able to find equilibrium shapes of the minicircles. We note that our coarse-grained models of minicircles are oblivious to their sequence and do not exploit information on sequence-dependent elastic properties. However, the uniform beam model of DNA elasticity presented here predicts shapes of DNA minicircles (shown in Figures 3A and 6) that compare favorably with the direct observations of 336 bp minicircles (13) (shown in Figure 4B).

Our theoretical model predicts a very weak dependence of the shapes of DNA minicircles with a few hundred base pairs on the aspect ratio *d*_*s*_/*L*, as shown in Figure 4A. According to the model, the change in Δ*Lk*, alone, gives sufficient information to describe basic features of the minicircle configuration: Δ*Lk* = 0 is an open circle; |Δ*Lk*| = ± 1 is at the transition between open circle and writhed conformations; |Δ*Lk*| = ±2 is multi-stable; and |Δ*Lk*| > 3 always exhibits self-contact (compare with the corresponding shapes in Figures 3A and 6). This result means that no matter whether the minicircle was 336 or 672 bp, the loss of three helical turns was enough to disallow the open circle conformation as an energy-minimal solution. These results suggest that it is the absolute value of Δ*Lk* and not the superhelical density σ = Δ*Lk*/*Lk*_0_ that governs the conformational landscape of small DNA minicircles (with the length having a small influence via the *d*_*s*_/*L* ratio). This finding provides an important input to future models of circularized polymers.

Incorporating torsional interactions is more difficult than just bending interactions and is often neglected when constructing models of circularized molecules (71–74). Because the torsional forces play a role even when Δ*Lk* is close to zero, care must be taken when generalizing such models for the context of supercoiled DNA. We have shown here that regardless of the length of the DNA molecule the torsional forces are of the same order of magnitude as pure bending forces.

While both bending and shearing forces remain comparable in magnitude even for longer minicircles, the relative importance of thermal fluctuations increases proportionally to the length. For the short minicircles considered here, thermal effects are not strong enough to push them out of their global energy minimum corresponding to the presented shapes. The results of Coleman and Swigon (42), however, show that mechanically stable branched plectonemic configurations exist that correspond to local energy minima of higher elastic energy. These high energy states can be accessed provided that the forces from thermal motion exceed the elastic forces, which is the case for minicircles of lengths much longer than the persistence length. For the minicircles studied here, we assumed that the minimal elastic energy shape was rigidly maintained throughout an experiment. To predict hydrodynamic properties of longer DNA circles, prone to conformational flexibility due to Brownian effects, one has to use different modeling techniques, e.g., such as those inspired by models of intrinsically disordered proteins (75).

The elastic-energy minimizing centerline shapes were found for minicircles with different Δ*Lk* with hydrodynamic thickness determined by calibration based on AUC experimental data. These shapes were then used to construct hydrodynamic models to compute *R*_*h*_, which, in turn, was used to compare the theoretical diffusion and sedimentation coefficients with the AUC measurement. The comparison (Table 2) shows general agreement, which is satisfactory given the simplicity of the underlying coarse-grained model. These results confirm the predictive capabilities of uniform elasticity models, combined with a hydrodynamic calculations package (ZENO) to interpret and guide AUC measurements. We expect that the proposed modeling strategy could be beneficially employed to similar problems in the dynamics of DNA and perhaps extended further to account for sequence-specific effects and modified intramolecular interactions. One way forward would be to introduce local, sequence-dependent elasticity by allowing the bending stiffness of the elastic beam model to vary along the centerline of the minicircle. This extension should aid the studies of localized kinking, seen in earlier experiments (61), and the formation of bubbles in DNA minicircles under negative supercoiling (12).

This work represents significant progress in understanding and modeling the sedimentation of a biological molecule with a complex and dynamic conformation. Sedimentation of roughly spherical molecules (e.g., many proteins) is fairly well understood, but linear and minicircle DNA do not adopt a spherical conformation. Using 336 bp minicircles as a model system we are now able, for the first time, to fully test the theoretical models. We anticipate that this testing will allow us to further improve these models and expand the use of AUC to include a larger repertoire of important and complex biological molecules. We have successfully applied two stage modeling (combination of energy minimization to find the shape then applying hydrodynamic modeling for rigid configurations) to find hydrodynamic properties of a DNA molecule. Our approach developed here yielding the prediction of shape and hydrodynamic properties is applicable to other complex biomolecules.

## CONCLUSION

The work presented here is a step toward understanding how supercoiling and curvature affect DNA shape and hydrodynamic properties, which in turn affect important DNA activities. The DNA solvation shell and counterions as well as DNA shape all affect DNA structure to influence how the DNA code is protected, accessed, modified, and activated. It will take a combination of approaches to fully understand this remarkable molecule.

## DATA AVAILABILITY

The UltraScan software used to analyze the AUC data is open source and freely available from Github repository. The AUC data itself is available upon request from the UltraScan LIMS server at the Canadian Center for Hydrodynamics.

The algorithm for finding minimal energy shapes, initial conditions, and final configurations can be found in the Zenodo repository 7501673 (76).

*Lk*-*Wr* pairs computed from energy minimal shapes as well as hydrodynamic radii computed using the ZENO software can be found in the Zenodo repository Zenodo repository 7501675 (77).

## Supplementary Material

gkad183_Supplemental_File

## References

[1] Franklin R.E. , Gosling R.G. Molecular configuration in sodium thymonucleate. Nature. 1953; 171:740–741. 13054694 10.1038/171740a0

[2] Lieberman-Aiden E. , van Berkum N.L., Williams L., Imakaev M., Ragoczy T., Telling A., Amit I., Lajoie B.R., Sabo P.J., Dorschner M.O. et al. Comprehensive mapping of long-range interactions reveals folding principles of the human genome. Science. 2009; 326:289–293. 19815776 10.1126/science.1181369 PMC2858594

[3] Dorman C.J. Genome architecture and global gene regulation in bacteria: making progress towards a unified model?. Nat. Rev. Microbiol. 2013; 11:349–355. 23549066 10.1038/nrmicro3007

[4] Fisher J.K. , Bourniquel A., Witz G., Weiner B., Prentiss M., Kleckner N. Four-dimensional imaging of E. coli nucleoid organization and dynamics in living cells. Cell. 2013; 153:882–895. 23623305 10.1016/j.cell.2013.04.006 PMC3670778

[5] Kleckner N. , Fisher J.K., Stouf M., White M.A., Bates D., Witz G. The bacterial nucleoid: nature, dynamics and sister segregation. Curr. Opin. Microbiol. 2014; 22:127–137. 25460806 10.1016/j.mib.2014.10.001 PMC4359759

[6] Dorman C.J. DNA supercoiling and transcription in bacteria: a two-way street. BMC Mol. Cell Biol. 2019; 20:26. 31319794 10.1186/s12860-019-0211-6 PMC6639932

[7] Baranello L. , Levens D., Gupta A., Kouzine F. The importance of being supercoiled: How DNA mechanics regulate dynamic processes. Biochimica et Biophysica Acta (BBA) - Gene Regulatory Mechanisms. 2012; 1819:632–638. 22233557 10.1016/j.bbagrm.2011.12.007 PMC3354648

[8] Magnan D. , Bates D. Regulation of DNA replication initiation by chromosome structure. J. Bacteriol. 2015; 197:3370–3377. 26283772 10.1128/JB.00446-15 PMC4621074

[9] Drew H.R. , Weeks J.R., Travers A.A. Negative supercoiling induces spontaneous unwinding of a bacterial promoter. EMBO J. 1985; 4:1025–1032. 2990904 10.1002/j.1460-2075.1985.tb03734.x PMC554295

[10] Kim S.H. , Ganji M., Kim E., van der Torre J., Abbondanzieri E., Dekker C. DNA sequence encodes the position of DNA supercoils. eLife. 2018; 7:e36557. 30523779 10.7554/eLife.36557 PMC6301789

[11] Levens D. How the c-myc promoter works and why it sometimes does not. J. Natl. Cancer Ins. Monograp. 2008; 39:41–43. 10.1093/jncimonographs/lgn004 PMC2683261 18648001

[12] Fogg J.M. , Judge A.K., Stricker E., Chan H.L., Zechiedrich L. Supercoiling and looping promote DNA base accessibility and coordination among distant sites. Nat. Commun. 2021; 12:5683. 34584096 10.1038/s41467-021-25936-2 PMC8478907

[13] Irobalieva R.N. , Fogg J.M., Catanese D.J., Sutthibutpong T., Chen M., Barker A.K., Ludtke S.J., Harris S.A., Schmid M.F., Chiu W. et al. Structural diversity of supercoiled DNA. Nat. Commun. 2015; 6:8440. 26455586 10.1038/ncomms9440 PMC4608029

[14] Fogg J.M. , Randall G.L., Pettitt B.M., Sumners D. W.L., Harris S.A., Zechiedrich L. Bullied no more: when and how DNA shoves proteins around. Quart. Rev. Biophys. 2012; 45:257–299. 10.1017/S0033583512000054 PMC4866820 22850561

[15] Bond L.M. , Peters J.P., Becker N.A., Kahn J.D., Maher L.J. Gene repression by minimal lac loops in vivo. Nucleic Acids Res. 2010; 38:8072–8082. 21149272 10.1093/nar/gkq755 PMC3001091

[16] Czapla L. , Grosner M.A., Swigon D., Olson W.K. Interplay of protein and DNA structure revealed in simulations of the lac operon. PLoS ONE. 2013; 8:e56548. 23457581 10.1371/journal.pone.0056548 PMC3572996

[17] Garcia H.G. , Grayson P., Han L., Inamdar M., Kondev J., Nelson P.C., Phillips R., Widom J., Wiggins P.A. Biological consequences of tightly bent DNA: The other life of a macromolecular celebrity. Biopolymers. 2007; 85:115–130. 17103419 10.1002/bip.20627 PMC3496788

[18] Luger K. , Mäder A.W., Richmond R.K., Sargent D.F., Richmond T.J. Crystal structure of the nucleosome core particle at 2.8 Å resolution. Nature. 1997; 389:251–260. 9305837 10.1038/38444

[19] Pyne A.L.B. , Noy A., Main K.H.S., Velasco-Berrelleza V., Piperakis M.M., Mitchenall L.A., Cugliandolo F.M., Beton J.G., Stevenson C. E.M., Hoogenboom B.W. et al. Base-pair resolution analysis of the effect of supercoiling on DNA flexibility and major groove recognition by triplex-forming oligonucleotides. Nat. Commun. 2021; 12:1053. 33594049 10.1038/s41467-021-21243-y PMC7887228

[20] Basu A. , Bobrovnikov D.G., Qureshi Z., Kayikcioglu T., Ngo T.T.M., Ranjan A., Eustermann S., Cieza B., Morgan M.T., Hejna M. et al. Measuring DNA mechanics on the genome scale. Nature. 2021; 589:462–467. 33328628 10.1038/s41586-020-03052-3 PMC7855230

[21] Li K. , Carroll M., Vafabakhsh R., Wang X.A., Wang J.-P. DNAcycP: a deep learning tool for DNA cyclizability prediction. Nucleic Acids Res. 2022; 50:3142–3154. 35288750 10.1093/nar/gkac162 PMC8989542

[22] Demeler B. , Gorbet G.E. Uchiyama S. , Arisaka F., Stafford W.F., Laue T. Analytical ultracentrifugation data analysis with UltraScan-III. Anal. Ultracentr. 2016; Tokyo SpringerJapan 119–143.

[23] Fogg J.M. , Kolmakova N., Rees I., Magonov S., Hansma H., Perona J.J., Zechiedrich E.L. Exploring writhe in supercoiled minicircle DNA. J. Phys. Cond. Matt. 2006; 18:S145–S159. 10.1088/0953-8984/18/14/S01 PMC2662687 19337583

[24] Fuller F.B. Decomposition of the linking number of a closed ribbon: A problem from molecular biology. Proc. Natl. Acad. Sci. 1978; 75:3557–3561. 16592550 10.1073/pnas.75.8.3557 PMC392823

[25] Savelyev A. , Gorbet G.E., Henrickson A., Demeler B. Moving analytical ultracentrifugation software to a good manufacturing practices (GMP) environment. PLOS Comput. Biol. 2020; 16:e1007942. 32559250 10.1371/journal.pcbi.1007942 PMC7347214

[26] Cao W. , Demeler B. Modeling analytical ultracentrifugation experiments with an adaptive space-time finite element solution of the Lamm equation. Biophys. J. 2005; 89:1589–1602. 15980162 10.1529/biophysj.105.061135 PMC1366663

[27] Cao W. , Demeler B. Modeling analytical ultracentrifugation experiments with an adaptive space-time finite element solution for multicomponent reacting systems. Biophys. J. 2008; 95:54–65. 18390609 10.1529/biophysj.107.123950 PMC2426643

[28] Brookes E. , Cao W., Demeler B. A two-dimensional spectrum analysis for sedimentation velocity experiments of mixtures with heterogeneity in molecular weight and shape. Eur. Biophys. J. 2010; 39:405–414. 19247646 10.1007/s00249-009-0413-5 PMC12146834

[29] Demeler B. Methods for the design and analysis of sedimentation velocity and sedimentation equilibrium experiments with proteins. Curr. Protoc. Protein Sci. 2010; 60:7.13.1–7.13.24. 10.1002/0471140864.ps0713s60 PMC4547541 20393977

[30] Brookes E.H. , Demeler B. Parsimonious regularization using genetic algorithms applied to the analysis of analytical ultracentrifugation experiments. Proceedings of the 9th annual conference on genetic and evolutionary computation - GECCO ’07, London. 2007; 361–368.

[31] Demeler B. , Brookes E. Monte Carlo analysis of sedimentation experiments. Coll. Pol. Sci. 2008; 286:129–137.

[32] Williams T.L. , Gorbet G.E., Demeler B. Multi-speed sedimentation velocity simulations with UltraScan-III. Eur. Biophys. J. 2018; 47:815–823. 29748855 10.1007/s00249-018-1308-0 PMC6158090

[33] Gorbet G.E. , Mohapatra S., Demeler B. Multi-speed sedimentation velocity implementation in UltraScan-III. Eur. Biophys. J. 2018; 47:825–835. 29610996 10.1007/s00249-018-1297-z PMC6676492

[34] Eisenberg H. Modern analytical ultracentrifugation in protein science: Look forward, not back. Prot. Sci. 2003; 12:2647–2649. 10.1110/ps.0235803 PMC2366943 14573877

[35] Van Holde K.E. 1985;

[36] Durchschlag H. Hinz H.-J. Specific volumes of biological macromolecules and some other molecules of biological interest. Thermodynamic data for Biochemistry and Biotechnology. 1986; Berlin, Heidelberg Springer Verlag.

[37] Durchschlag H. Determination of the partial specific volume of conjugated proteins. Coll. Pol. Sci. 1989; 267:1139–1150.

[38] Korolev N. , Lyubartsev A.P., Rupprecht A., Nordenskiöld L. Competitive Binding of Mg^2 +^, Ca^2 +^, Na^+^, and K^+^ Ions to DNA in Oriented DNA Fibers: Experimental and Monte Carlo Simulation Results. Biophys. J. 1999; 77:2736–2749. 10545373 10.1016/s0006-3495(99)77107-9 PMC1300547

[39] Landau L.D. , Lifshitz E.M. Theory of elasticity, Elsevier, Butterworth-Heinemann. 2009; Amsterdam.

[40] Călugăreanu G. L’intégrale de Gauss et l’Analyse des nœuds tridimensionnels. Revue Roumaine de Mathématiques Pures et Appliquées. 1959; 4:5–20.

[41] Bates A.D. , Maxwell A. DNA topology. 2005; Oxford Oxford University Press.

[42] Coleman B.D. , Swigon D. Theory of supercoiled elastic rings with self-contact and its application to DNA plasmids. J. Elas. 2000; 60:173–221.

[43] Zajac E.E. Stability of two planar loop elasticas. J. Appl. Mech. 1962; 29:136–142.

[44] Levitt M. Protein folding by restrained energy minimization and molecular dynamics. J. Mol. Biol. 1983; 170:723–764. 6195346 10.1016/s0022-2836(83)80129-6

[45] Johnson R.E. , Wu T.Y. Hydromechanics of low-Reynolds-number flow. Part 5. Motion of a slender torus. J. Fluid Mech. 1979; 95:263–277.

[46] Goren S.L. , O’Neill M.E. Asymmetric creeping motion of an open torus. J. Fluid Mech. 1980; 101:97–110.

[47] Juba D. , Audus D.J., Mascagni M., Douglas J.F., Keyrouz W. ZENO: Software for calculating hydrodynamic, electrical, and shape properties of polymer and particle suspensions. J. Res. Natl. Ins. Stand. Tech. 2017; 122:20. 10.6028/jres.122.020 PMC7339781 34877114

[48] Hubbard J.B. , Douglas J.F. Hydrodynamic friction of arbitrarily shaped Brownian particles. Phys. Rev. E. 1993; 47:R2983–R2986. 10.1103/physreve.47.r2983 9960451

[49] Rybenkov V.V. , Vologodskii A.V., Cozzarelli N.R. The effect of ionic conditions on DNA helical repeat, effective diameter and free energy of supercoiling. Nucleic Acids Res. 1997; 25:1412–1418. 9060437 10.1093/nar/25.7.1412 PMC146597

[50] Xu Y.C. , Bremer H. Winding of the DNA helix by divalent metal ions. Nucleic Acids Res. 1997; 25:4067–4071. 9321659 10.1093/nar/25.20.4067 PMC147022

[51] Pak A.J. , Voth G.A. Advances in coarse-grained modeling of macromolecular complexes. Cur. Opin. Struct. Biol. 2018; 52:119–126. 10.1016/j.sbi.2018.11.005 PMC6296860 30508766

[52] de la Torre J.G. , Bloomfield V.A. Hydrodynamic properties of complex, rigid, biological macromolecules: theory and applications. Quart. Rev. Biophys. 1981; 14:81–139. 10.1017/s0033583500002080 7025081

[53] Hückel E. , Debye P. The theory of electrolytes: I. Lowering of freezing point and related phenomena. Physikalische Zeitschrift. 1923; 24:185–206.

[54] Lim S. , Kim Y., Swigon D. Dynamics of an electrostatically charged elastic rod in fluid. Proc. Roy. Soc. A: Math., Phys. Eng. Sci. 2011; 467:569–590.

[55] Robertson R.M. , Laib S., Smith D.E. Diffusion of isolated DNA molecules: Dependence on length and topology. Proc. Natl. Acad. Sci. 2006; 103:7310–7314. 16648255 10.1073/pnas.0601903103 PMC1450111

[56] Mantelli S. , Muller P., Harlepp S., Maaloum M. Conformational analysis and estimation of the persistence length of DNA using atomic force microscopy in solution. Soft Matt. 2011; 7:3412.

[57] Odijk T. Polyelectrolytes near the rod limit. J. Poly. Sci.: Poly. Phys. Edn. 1977; 15:477–483.

[58] Skolnick J. , Fixman M. Electrostatic persistence length of a wormlike polyelectrolyte. Macromolecules. 1977; 10:944–948.

[59] Manning G.S. Counterion condensation on a helical charge lattice. Macromolecules. 2001; 34:4650–4655.

[60] Manning G.S. The persistence length of DNA is reached from the persistence length of its null isomer through an internal electrostatic stretching force. Biophys. J. 2006; 91:3607–3616. 16935960 10.1529/biophysj.106.089029 PMC1630458

[61] Lionberger T.A. , Demurtas D., Witz G., Dorier J., Lillian T., Meyhöfer E., Stasiak A. Cooperative kinking at distant sites in mechanically stressed DNA. Nucleic Acids Res. 2011; 39:9820–9832. 21917856 10.1093/nar/gkr666 PMC3239204

[62] Wang Q. , Irobalieva R.N., Chiu W., Schmid M.F., Fogg J.M., Zechiedrich L., Pettitt B.M. Influence of DNA sequence on the structure of minicircles under torsional stress. Nucleic Acids Res. 2017; 45:7633–7642. 28609782 10.1093/nar/gkx516 PMC5737869

[63] Johnson R.E. An improved slender-body theory for Stokes flow. J. Fluid Mech. 1980; 99:411–431.

[64] Adamczyk Z. , Sadlej K., Wajnryb E., Ekiel-Jeżewska M.L., Warszyński P. Hydrodynamic radii and diffusion coefficients of particle aggregates derived from the bead model. J. Colloid Interface Sci. 2010; 347:192–201. 20430400 10.1016/j.jcis.2010.03.066

[65] Fernandes M.X. Calculation of hydrodynamic properties of small nucleic acids from their atomic structure. Nucleic Acids Res. 2002; 30:1782–1788. 11937632 10.1093/nar/30.8.1782 PMC113193

[66] Penkova N.A. , Sharapov M.G., Penkov N.V. Hydration shells of DNA from the point of view of terahertz time-domain spectroscopy. Int. J. Mol. Sci. 2021; 22:11089. 34681747 10.3390/ijms222011089 PMC8538832

[67] Laage D. , Elsaesser T., Hynes J.T. Water dynamics in the hydration shells of biomolecules. Chem. Rev. 2017; 117:10694–10725. 28248491 10.1021/acs.chemrev.6b00765 PMC5571470

[68] Stellwagen N.C. Electrophoresis of DNA in agarose gels, polyacrylamide gels and in free solution. Electrophoresis. 2009; 30:S188–S195. 19517510 10.1002/elps.200900052 PMC2757927

[69] Ziraldo R. , Shoura M.J., Fire A.Z., Levene S.D. Deconvolution of nucleic-acid length distributions: a gel electrophoresis analysis tool and applications. Nucleic Acids Res. 2019; 47:e92–e92. 31226202 10.1093/nar/gkz534 PMC6895257

[70] Iubini S. , Orlandini E., Michieletto D., Baiesi M. Topological sieving of rings according to their rigidity. ACS Macro Lett. 2018; 7:1408–1412. 35651235 10.1021/acsmacrolett.8b00719

[71] Waszkiewicz R. , Szymczak P., Lisicki M. Stability of sedimenting flexible loops. J. Fluid Mech. 2021; 919:A14.

[72] Gruziel M. , Thyagarajan K., Dietler G., Stasiak A., Ekiel-Jeżewska M.L., Szymczak P. Periodic motion of sedimenting flexible knots. Phys. Rev. Lett. 2018; 121:127801. 30296142 10.1103/PhysRevLett.121.127801

[73] Gruziel-Słomka M. , Kondratiuk P., Szymczak P., Ekiel-Jeżewska M.L. Stokesian dynamics of sedimenting elastic rings. Soft Matter. 2019; 15:7262–7274. 31486465 10.1039/c9sm00598f

[74] Gruziel-Słomka M. , Kondratiuk P., Szymczak P., Ekiel-Jeżewska M.L. Correction: Stokesian dynamics of sedimenting elastic rings. Soft Matter. 2022; 18:4811–4811. 35708072 10.1039/d2sm90079c

[75] Cichocki B. , Rubin M., Niedzwiecka A., Szymczak P. Diffusion coefficients of elastic macromolecules. J. Fluid Mech. 2019; 878:R3.

[76] Waszkiewicz R. RadostW/twisted-loop: Manuscript version. 2023; Zenodo 10.5281/zenodo.7501673.

[77] Waszkiewicz R. RadostW/twisted-dna-results: Manuscript version. 2023; Zenodo 10.5281/zenodo.7501675.

