# Supplementary material for: DNA supercoiling-induced shapes alter minicircle hydrodynamic properties
Source: Nucleic Acids Res. 2023 Mar 27;51(8):4027–42. doi: 10.1093/nar/gkad183 (PMC10164573; doi:10.1093/nar/gkad183)

Supplementary Table 1: Topological composition of the DNA samples used. The composition of the samples was determined by quantification of fluorescently stained gels using image analysis software. N.D.: not detected.

Minicircle length, bp	Sample	Topoisomers present (% of total)	Trace species (% of total)
336	"Supercoiled"	$\Delta Lk = -3$ (48%)<br>$\Delta Lk = -2$ (41%)	$\Delta Lk = -1$ (7 %)<br>Nicked 336 bp (1 %)<br>Supercoiled 672 bp (3 %)
	Nicked	Nicked (93 %)	Nicked 672 bp (7 %)
	Relaxed	Relaxed (95 %)	Relaxed 672 bp (5 %)
	"Hypernegatively supercoiled"	$\Delta Lk = -6$ (61 %)<br>$\Delta Lk = -5$ (33 %)	Nicked 336 bp (4 %)<br>Supercoiled 672 bp (3 %)
	Linear	Linear (100 %)	N.D.
672	"Supercoiled"	$\Delta Lk = -4$ (63 %)<br>$\Delta Lk = -5$ (24 %)	$\Delta Lk = -6$ (6 %)<br>$\Delta Lk = -2$ (4 %)<br>$\Delta Lk = -3$ (2 %)<br>Nicked (2 %)
	Nicked	Nicked (100 %)	N.D.

Supplementary Figure 1: Electrophoretic mobility of minicircle DNA on agarose gels. DNA samples were analyzed by agarose gel electrophoresis in either a) 1.5 % agarose or b) 3 % agarose in the presence of 1 mM EDTA. Mr_1: 100 bp DNA ladder, lanes 2–6: 336 bp minicircle DNA samples (Sc: "supercoiled", N: nicked, R: relaxed, H: "hypernegatively supercoiled", L: linear), lanes 7–8: 672 bp DNA samples (Sc: "supercoiled", N: nicked), Mr_1: low molecular weight DNA ladder.

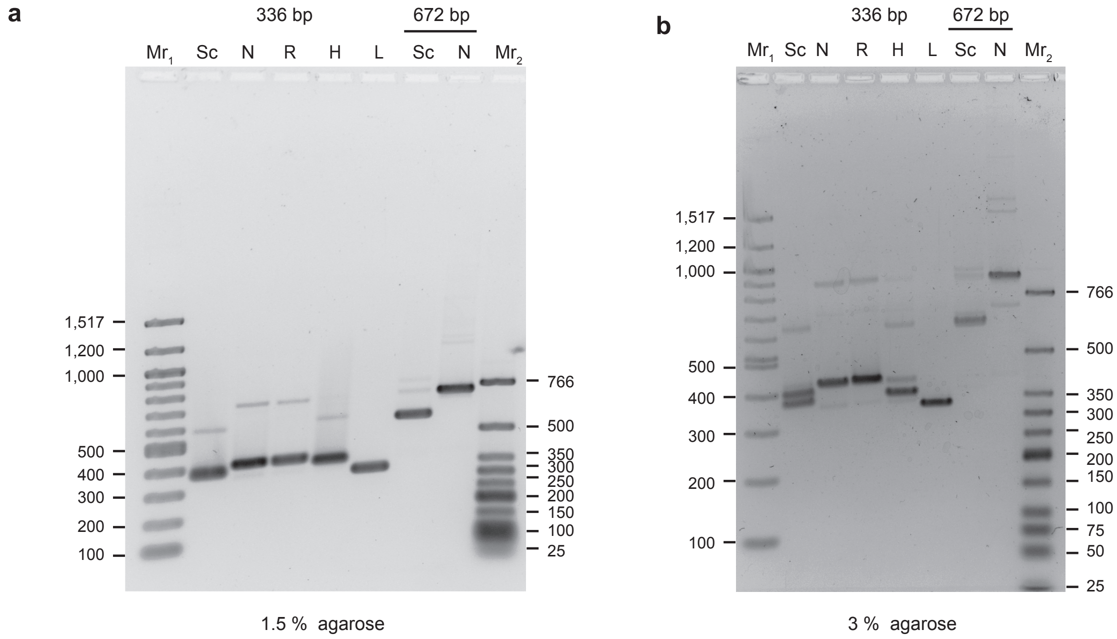

Supplement: gkad183_Supplemental_File [file gkad183_supplemental_file.pdf]
